# Analysis of spatial patellofemoral alignment using novel three-dimensional measurements based on weight-bearing cone-beam CT

**DOI:** 10.1186/s13244-024-01883-6

**Published:** 2025-01-02

**Authors:** Yurou Chen, Fan Yu, Fanzhuang Rong, Furong Lv, Fajin Lv, Jia Li

**Affiliations:** 1 Department of Radiology, The First Affiliated Hospital of Chongqing Medical University, Chongqing, China; 2 Shenzhen Angell Technology Co. Ltd., Shenzhen, China

**Keywords:** Three-dimensional measurement, Weight-bearing, Patellofemoral alignment, Recurrent patellar dislocation, Preoperative planning

## Abstract

**Objectives:**

To propose a reliable and standard 3D assessment method to analyze the effect of weight-bearing (WB) status on the location of patella and clarify the diagnostic performance of 3D parameters for recurrent patellar dislocation (RPD) in WB and non-weight-bearing (NWB) conditions.

**Methods:**

Sixty-five knees of RPD patients and 99 knees of controls were included. Eight landmarks, two lines and a coordinate system were defined on 3D bone models of knees based on weight-bearing CT and non-weight-bearing CT. The shift and tilt of patella in three orthogonal axes (*X*_shift_, *Y*_shift_, *Z*_shift_, *X*_tilt_, *Y*_tilt_, *Z*_tilt_) were evaluated.

**Results:**

*X*_shift_ and *Y*_shift_ were significantly higher, *Z*_shift_, *X*_tilt_ and *Y*_tilt_ were significantly lower in WB condition than NWB condition (*p* < 0.001, *p* < 0.001, *p* = 0.001, *p* = 0.002, *p* = 0.010). In both WB and NWB conditions, *X*_shift_, *Y*_shift_ and *Z*_tilt_ were significantly higher, and *X*_tilt_ was significantly lower in the RPD group than the control group (WB/NWB: *p* < 0.001/*p* = 0.002, *p* < 0.001/*p* = 0.001, *p* < 0.001/*p* < 0.001, *p* < 0.001/*p* = 0.009). In WB condition, *Z*_shift_ and *Y*_tilt_ were significantly higher in the RPD group than the control group (*p* = 0.011, *p* < 0.001). *Z*_tilt_ had the best diagnostic performance for RPD in both WB and NWB conditions, with AUC of 0.887 (95% CI: 0.828, 0.946) and 0.885 (95% CI: 0.822, 0.947), respectively.

**Conclusions:**

The 3D measurement method reliably and comprehensively reflected the relative spatial position relationship of the patellofemoral joint. It can be applied to the 3D preoperative planning of patellofemoral procedures. In addition, patellofemoral evaluation under the WB condition was essential to detect subtle underlying risk factors for RPD, with axial lateral patellar tilt being the best predictor.

**Critical relevance statement:**

This 3D measurement method under weight-bearing conditions contributes to comprehensively describing the relative spatial position of the patellofemoral joint in a standardized way and can be applied to preoperative evaluation for recurrent patellar dislocation.

**Key Points:**

- Patellofemoral alignment is a 3D problem, and the accuracy of 2D parameters has been questioned.
- 3D measurement was reliable and comprehensively reflected relative spatial relationships of the patellofemoral joint.
- 3D measurements under weight-bearing condition help preoperative evaluation for RPD.

**Graphical Abstract:**

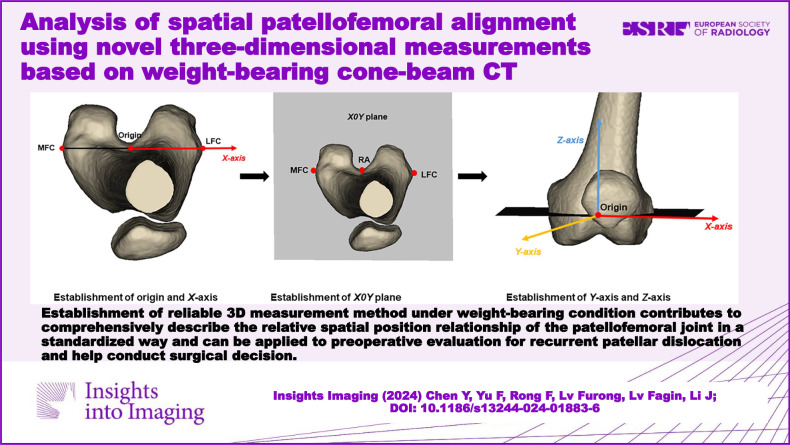

## Introduction

Patellar instability is a common event in orthopedics and tends to affect adolescents [1]. Current clinical preoperative examinations for recurrent patellar dislocation (RPD) include digital radiography (DR), computed tomography (CT), and magnetic resonance imaging (MRI) [2]. Conventional CT and MRI examinations are performed with the patient in supine position and the lower limb muscles relaxed, which avoids the factor of quadriceps contraction in the weight-bearing (WB) conditions that tends to cause patellar instability [3–5]. Studies revealed that patellofemoral alignment varied with quadriceps contraction [6] and loading [7, 8]. A new weight-bearing cone-beam CT (WBCT) has been developed to obtain high-resolution three-dimensional (3D) data of lower limbs in a single rotation in full physiological WB condition to more accurately reflect the patellofemoral alignment in its functional state [9, 10]. WBCT provided excellent image quality for bone visualization and adequate image quality for soft tissue visualization tasks [11]. In addition, the radiation dose of WBCT is significantly lower than that of conventional CT [12].

Despite the development of imaging technology, the correlation between preoperative clinical evaluation of patellofemoral alignment and postoperative outcomes remains puzzling. This is attributed to the high variability of existing measurement methods. Numerous two-dimensional parameters performed with DR or projection images of CT and MRI have been proposed [6, 13–17]. Determination of anatomical landmarks based on 2D plane is influenced by the position of the lower extremity during scanning, slice thickness, and slice selection [18, 19]. Therefore, even the evaluation of the same subject obtained from different scans could differ considerably. Moreover, multiple parameters have been proposed to describe one risk factor, such as Insall-Salvati, Caton-Deschamps, Blackburne-Peel, and modified Insall-Salvati for describing patellar height. Measurement methods are defined differently for each parameter, making it difficult to achieve a truly uniform comparison of the results of studies that use different parameters. There are even contradictions between different parameters in the same study [20]. Standardized clinical quantification of the patellofemoral alignment remains undefined. Even though gross patellofemoral malalignment is easy to identify, the detection of subtle underlying risk factors for patellofemoral instability needs to be improved. Inaccurate preoperative quantification is also a huge obstacle for clinicians to make appropriate surgical decisions. An increasing number of researchers have focused on 3D patellofemoral analysis based on 3D bone models rather than projections [21–23]. It is worthwhile to propose a standardized 3D measurement method to accurately describe the spatial position of the complex patellofemoral joint.

The present study proposes a coordinate system based on 3D data of the knee obtained by WBCT and non-weight-bearing CT (NWBCT) to (1) enable a reliable 3D assessment method that comprehensively reflects the spatial patellofemoral alignment, (2) analyze the effect of WB status on the position of the patella, and (3) clarify the diagnostic performance of 3D parameters for RPD in WB and non-weight-bearing (NWB) conditions. It is hypothesized that when the 3D measurement method for patellofemoral joint was reliable, the measurements would be significantly different between the WB and NWB conditions.

## Materials and methods

### Participants

This prospective study was approved by the Committee for Human Research of our institution (No. 2023-139). Participants were recruited consecutively from January 2023 to September 2023. Two senior orthopedic surgeons took a history and conducted the physical examinations to include and exclude all the patients and controls. The RPD group included patients who have experienced at least two dislocations. The exclusion criteria were as follows: (1) prior knee surgery; (2) osteoarthritis (≥ Kellgren-Lawrence grade 3). For ethical reasons, the control group included patients requiring lower limb CT scans, such as those with lower limb injuries, benign tumors, etc. The healthy sides were included in the control group, and the affected sides were also included when there was no involvement of the patellofemoral joint. The exclusion criteria were the same as those used to exclude RPD patients.

### WBCT protocol

**Fig. 1:**
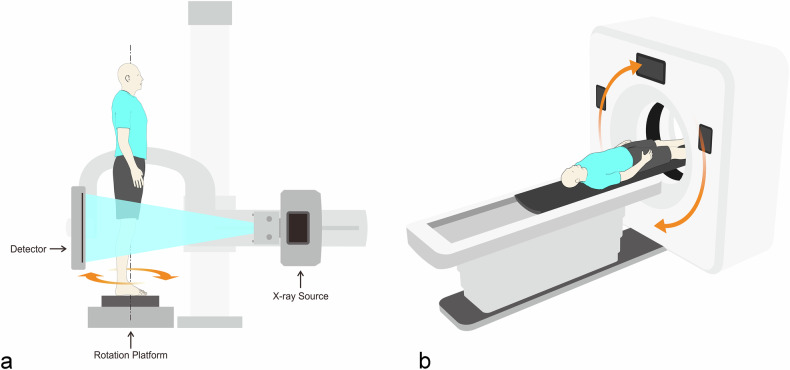
**a** Examination of weight-bearing CT. **b** Examination of non-weight-bearing CT

The WBCT device (DTP580B-3 Angell Technology, Shenzhen, China) has a motorized standing platform between the X-ray tube and flat panel detector. During the scanning, the subject stood with the lower limbs fully extended and was uniformly rotated by the motorized platform for 3D data acquisition. The imaging protocol was as follows: display field of view 300 × 300 × 300 mm, reconstruction thickness 1 mm, reconstruction slice spacing 1 mm, tube output 110 kV and 5 mA, source to image distance 120 cm, scan time 29 s (Fig. 1a).

### NWBCT protocol

The NWBCT images were acquired using conventional CT (Aquilion ONE, Canon Medical Systems, Otawara, Japan). The subject was in supine position with the lower limbs fully extended and relaxed. Imaging protocol was as follows: display field of view 250 × 250 × 160 mm, slice thickness 0.5 mm, slice spacing 0.5 mm, tube rotation 0.5 s, and tube output 100 kV and 70 mA (Fig. 1b).

### 3D measurement

Two fellowship-trained musculoskeletal radiologists (A and B, with 2 and 9 years of clinical experience, respectively) independently performed the 3D measurements after all the data of the two groups had been mixed blindly and randomly distributed. Furthermore, all measurements were repeated by one of the radiologists (B), with an interval between the first and second measurements of at least 4 weeks.

### Generation of 3D bone models

Images in DICOM format were imported into Mimics software (21.0, Materialise, Haasrode, Belgium) to create 3D bone models of knees.

### Definition of bony landmarks and lines

Landmarks were defined in Mimics and both 3D models and CT images were used to increase precision of landmark location.

**Fig. 2:**
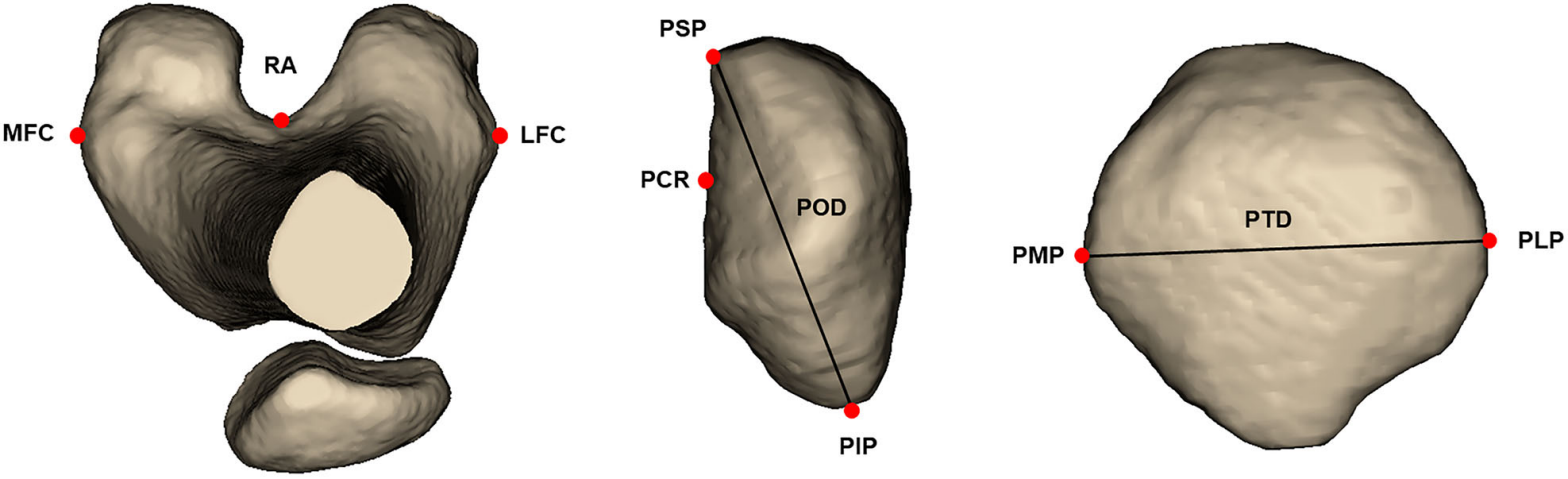
Definition of bony landmarks and lines. Medial femoral condyle (MFC): the most prominent point on the medial facet of the femoral condyle; lateral femoral condyle (LFC): the most prominent point on the lateral facet of the femoral condyle; Roman arch (RA): the deepest point of the classic Roman arch; patellar superior point (PSP): the most superior point of the patella; patellar inferior point (PIP): the most inferior point of the patella; patellar medial point (PMP): the most medial point of the patella; patellar lateral point (PLP): the most lateral point of the patella; patellar central ridge (PCR): the central ridge of the patella; patellar oblique diameter (POD): the line between PSP and PIP; patellar transverse diameter (PTD): the line between PMP and PLP

Medial femoral condyle (MFC), lateral femoral condyle (LFC), Roman arch (RA) [24], patellar superior point (PSP), patellar inferior point (PIP), patellar medial point (PMP), patellar lateral point (PLP), patellar central ridge (PCR), patellar oblique diameter (POD) and patellar transverse diameter (PTD) were defined (Fig. 2).

### Establishment of coordinate system

**Fig. 3:**
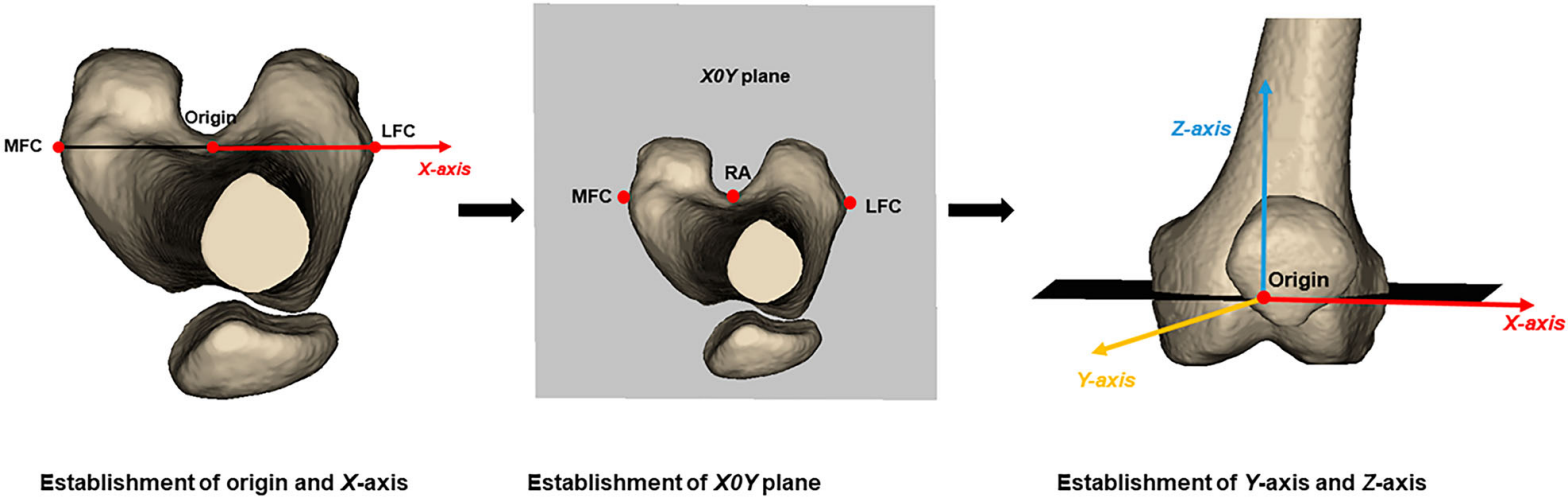
Establishment of coordinate system

The midpoint of MFC and LFC was defined as the origin. The line between MFC and LFC from the medial to lateral direction was defined as the *X-*axis. MFC, LFC and RA made up the *X0Y* plane. The normal vector of the *X0Y* plane over the origin from the inferior to superior direction was set as the *Z*-axis. The *Y*-axis from the posterior to anterior direction was set to fit the rule of a Cartesian coordinate system of 3D space (Fig. 3).

### 3D measurement of patellar position

The 3D models, landmarks, and lines were imported into a 3-matic module of Mimics. All the measurements were performed based on user-defined coordinate system.

The spatial displacement of the patella relative to the femur was simplified to shift of the center of the patellar ridge relative to the origin on three axes (*X*_shift_, *Y*_shift_, *Z*_shift_). Lateral, anterior and superior displacement were designated as positive, while medial, posterior and inferior as negative.

**Fig. 4:**
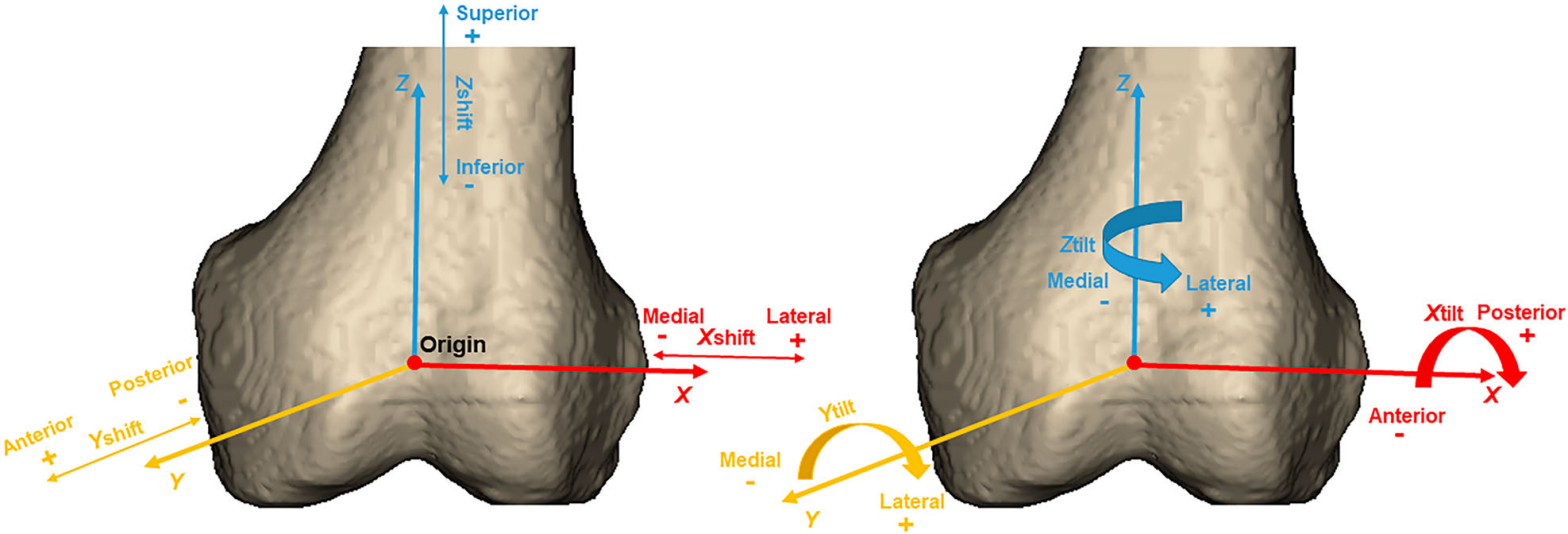
Spatial shift and tilt of the patella on three orthogonal axes

Tilt of the patella around the *X*-axis (*X*_tilt_) was defined as the angle between POD and *X0Z* plane, and posterior tilt was designated as positive. Tilt around the *Y*-axis (*Y*_tilt_) was defined as the angle between PTD and *X0Y* plane, and lateral tilt was designated as positive. Tilt around the *Z*-axis (*Z*_tilt_) was defined as the angle between PTD and *X0Z* plane, and lateral tilt was designated as positive (Fig. 4).

### Statistical analysis

SPSS Version 26.0 (IBM Corp., Armonk, NY, USA) was used for data analysis. Demographic data were analyzed using descriptive statistics. The single-measure intraclass correlation coefficient (ICC) was used to evaluate the inter-observer and intra-observer reliability (> 0.75, excellent; 0.4–0.75, fair to good; < 0.4, poor). Continuous variables with a normal distribution are expressed as mean ± standard deviations, or medians and percentiles were given if they are not normally distributed. Comparison between the RPD group and control group was evaluated with the Student’s *t*-test or Mann-Whitney *U* test, whichever was applicable. Comparison between the WB and NWB conditions was evaluated with the paired *t*-test or Wilcoxon signed-rank test, whichever was applicable. Receiver operating characteristic (ROC) curve presenting sensitivity and specificity was calculated for measurements, and the area under the ROC curve (AUC) was produced to assess the diagnostic accuracy of measurements for RPD. The reference ranges for non-normally distributed data are delimited by the 2.5th percentile and 97.5th percentile, and for normally distributed data by mean + 1.96 × standard deviation and mean − 1.96 × standard deviation. The level of significance was set at *p* < 0.05. Post hoc sample size calculation was performed using GPower software (version 3.1.9.6), and power of > 0.8 for all measurements was calculated.

## Results

### Demographic characteristics

A total of 65 knees were included in the RPD group, and 99 knees were included in the control group. All the participants underwent WBCT examination. Of these, 52 knees in the RPD group and 58 knees in the control group underwent both WBCT and NWBCT (Table 1).

**Table 1:** Characteristics of participants

	Undergo WBCT		Undergo both WBCT and NWBCT	
	RPD	Control	RPD	Control
Number of patients (knees)	33 (65)	52 (99)	28 (52)	31 (58)
Gender				
Male	6	18	6	11
Female	27	34	22	20
Age range (mean ± SD)	11–54 (25.97 ± 10.18)	17–83 (40.60 ± 15.63)	11–54 (25.57 ± 10.80)	17–67 (38.13 ± 14.92)
Side				
Left	32	51	25	30
Right	33	48	27	28

*NWBCT* non-weight-bearing cone-beam computed tomography, *RPD* recurrent patellar dislocation, *WBCT* weight-bearing cone-beam computed tomography

### Inter- and intra-observer reliability

The inter- and intra-observer reliability of all measurements was good to excellent, 0.768–0.981 and 0.844–0.962, respectively (Supplementary material 1).

## Radiological evaluation

### Comparison between the WB and NWB conditions

A total of 110 knees from the RPD group (52 knees) and the control group (58 knees) that underwent both WBCT and NWBCT were compared. *X*_shift_ and *Y*_shift_ were significantly higher in the WB condition than in the NWB condition (*p* < 0.001, *p* < 0.001); *Z*_shift_, *X*_tilt_ and *Y*_tilt_ were significantly lower in the WB condition (*p* = 0.001, *p* = 0.002, *p* = 0.010). There was no significant difference in *Z*_tilt_ between the WB and NWB conditions (Table 2).

**Table 2:** Comparison between the WB and NWB conditions in subjects including both RPD patients and controls

	WB (*n* = 110 knees)	NWB (*n* = 110 knees)	*p*-value
*X*_shift_ (mm)	3.34 (0.48–6.23)	1.05 (−0.83–3.69)	< 0.001
*Y*_shift_ (mm)	38.25 ± 4.70	36.40 ± 3.74	< 0.001
*Z*_shift_ (mm)	9.27 (6.19–14.9)	12.88 (8.69–15.98)	0.001
*X*_tilt_ (°)	12.09 (9.60–14.98)	13.86 (9.62–16.89)	0.002
*Y*_tilt_ (°)	0.04 ± 4.26	0.91 ± 4.03	0.010
*Z*_tilt_ (°)	11.76 (5.94–18.75)	12.15 (4.11–17.91)	0.116

*NWB* non-weight-bearing, *RPD* recurrent patellar dislocation, *WB* weight-bearing

### Comparison between the RPD group and control group

In both WB and NWB conditions, *X*_shift_, *Y*_shift_, and *Z*_tilt_ were significantly higher in the RPD group than in the control group (WB/NWB: *p* < 0.001/*p* = 0.002, *p* < 0.001/*p* = 0.001, *p* < 0.001/*p* < 0.001); *X*_tilt_ was significantly lower in the RPD group than in the control group (WB/NWB: *p* < 0.001/*p* = 0.009). In the WB condition, *Z*_shift_ and *Y*_tilt_ were significantly higher in the RPD group than in the control group (*p* = 0.011, *p* < 0.001). In the NWB condition, there were no significant differences in *Z*_shift_ and *Y*_tilt_ between the RPD and control groups (Table 3).

**Table 3:** Comparison between the RPD group and control group

	WB			NWB		
	RPD (*n* = 65 knees)	Control (*n* = 99 knees)	*p*-value	RPD (*n* = 52 knees)	Control (*n* = 58 knees)	*p*-value
*X*_shift_ (mm)	5.42 (3.19–7.83)	2.15 (−1.26–4.31)	< 0.001	3.51 (−0.31–6.29)	0.70 (−1.27–1.86)	0.002
*Y*_shift_ (mm)	39.26 ± 4.39	36.06 ± 4.34	< 0.001	37.34 (35.60–39.04)	34.53 (32.67–37.72)	0.001
*Z*_shift_ (mm)	12.33 (6.84–18.2)	8.74 (5.64–13.06)	0.011	13.65 ± 6.17	11.89 ± 4.74	0.097
*X*_tilt_ (°)	10.60 ± 5.43	13.67 ± 3.52	< 0.001	11.84 ± 5.59	14.38 ± 4.24	0.009
*Y*_tilt_ (°)	2.08 ± 4.14	−1.20 ± 4.05	< 0.001	1.90 (−1.02–3.63)	0.28 (−2.69–2.76)	0.079
*Z*_tilt_ (°)	21.06 ± 10.62	7.03 ± 5.83	< 0.001	17.89 (13.79–24.44)	5.33 (1.71–11.62)	< 0.001

*NWB* non-weight-bearing, *RPD* recurrent patellar dislocation, *WB* weight-bearing

### ROC analysis

*Z*_tilt_ had the best diagnostic performance for RPD in both WB and NWB conditions, with AUC of 0.887 (95% CI: 0.828, 0.946) and 0.885 (95% CI: 0.822, 0.947), respectively. The sensitivity, specificity and cutoff value of *Z*_tilt_ were 79.4%, 87.6% and 14.14 in WB condition, and 86%, 77.6% and 11.815 in NWB condition (Supplementary material 2).

3D patellofemoral measurements reference ranges are shown in Table 4.

**Table 4:** Reference ranges of controls in WB and NWB conditions

	Reference ranges	
	WB	NWB
*X*_shift_ (mm)	−6.61–8.18	−5.18–5.06
*Y*_shift_ (mm)	27.55–44.56	29.92–44.81
*Z*_shift_ (mm)	0.68–27.08	2.60–21.18
*X*_tilt_ (°)	6.78–20.56	6.07–22.69
*Y*_tilt_ (°)	−9.14–6.73	−7.33–8.50
*Z*_tilt_ (°)	−4.40–18.45	−3.41–21.24

*NWB* non-weight-bearing, *WB* weight-bearing

## Discussion

**Fig. 5:**
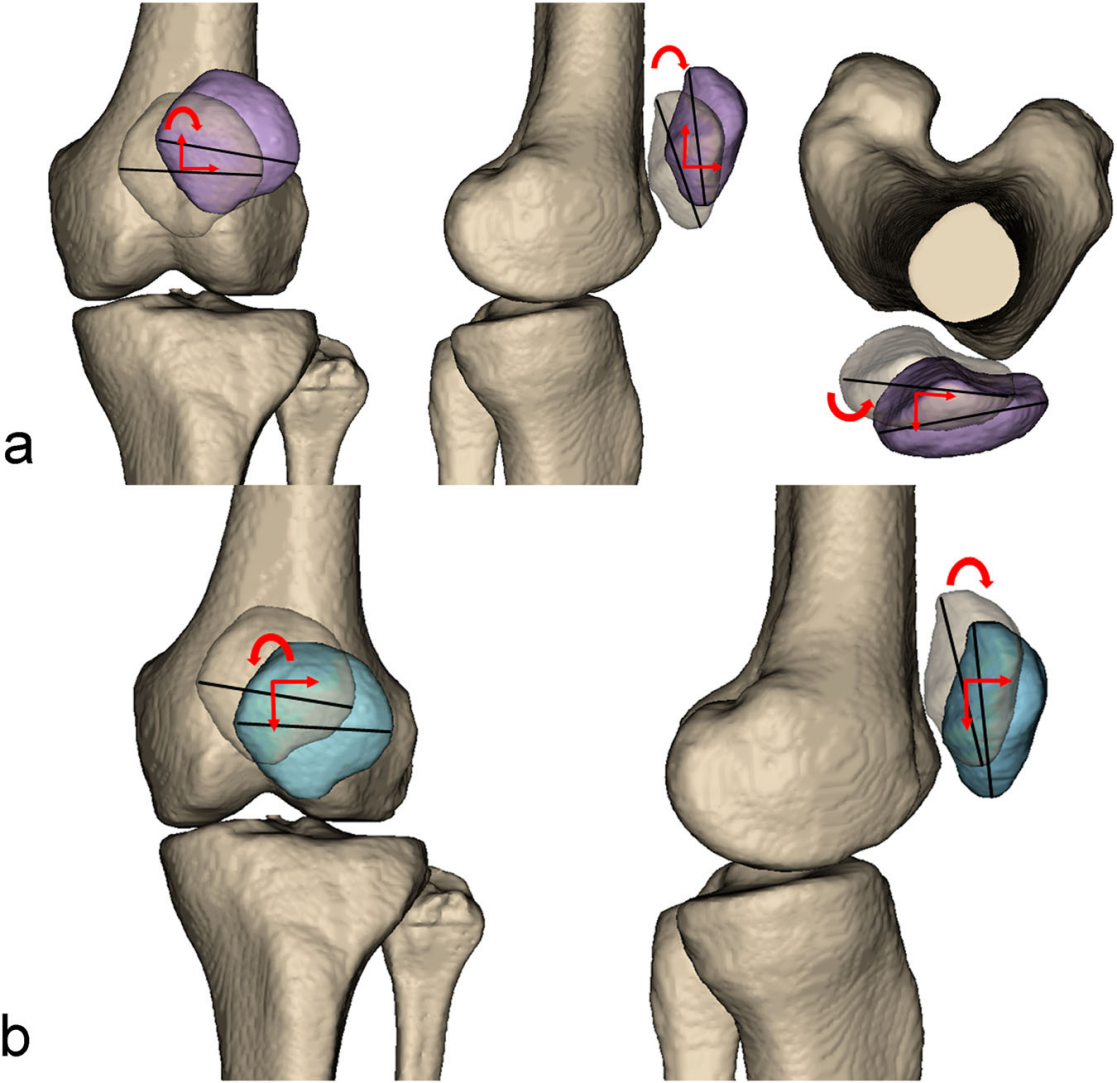
**a** Pattern of patellar position change in the RPD patients compared to the controls under the WB condition. **b** Pattern of patellar position changes under the WB condition compared to the NWB condition. NWB, non-weight-bearing; RPD, recurrent patellar dislocation; WB, weight-bearing

The key findings of this study were threefold. First, the new 3D patellofemoral measurement method was reliable and reflected the relative spatial position relationship of the patellofemoral joint. Second, the ability to detect subtle underlying risk factors for RPD based on WBCT is superior to that of NWBCT, and the axial lateral patellar tilt was the best predictor for RPD in the WB condition. Third, in the WB condition, patella in RPD patient was more laterally, anteriorly, and superiorly displaced, with less posteriorly tilt, more coronal lateral tilt, and more axial lateral tilt compared to the control group (Fig. 5a).

The present study proposed a new 3D patellofemoral measurement method. In order to make the patellofemoral joints of different individuals comparable, all knees were placed in the user-defined coordinate system established with the same procedure. The coordinate system was established with only femoral landmarks to avoid tibiofemoral torsion interference. Measurements of patellar position in three orthogonal axes reflect the relative spatial position relationship of the patellofemoral joint. Intra- and inter-observer reliability of all landmarks showed good to excellent, and through precise landmarks definition and a standardized coordinate system establishment procedure, intra- and inter-observer reliability of all measurements showed good to excellent. Compared with 2D parameters, 3D measurements in the present study were not limited to the choice of plane and patient positioning, and allowed for a comprehensive evaluation of the patellofemoral joint in a standardized way [25–29]. Considering the limited comparability of 3D measurements with 2D measurements, comparisons between 2D and 3D parameters were not performed in this study. The present study served as a pilot study with the primary aim of emphasizing the feasibility and reliability of the newly proposed 3D measurement method.

Accurate and reliable preoperative evaluation of the patellofemoral alignment is becoming increasingly important with the development of surgical techniques. In the study to predict the outcome of lateral retinacular release for anterior knee pain, Shea et al found that the effect of the position of the lower extremity (leg abduction or neutral) resulted in a significant difference in patellar tilt. This implied that normally aligned subjects could be diagnosed with excessive patellar tilt and thus inaccurately corrected if without standardized and reliable 3D measurements independent of lower limb positioning [30]. Therefore, the 3D measurement method can be used in the preoperative planning of patellar dislocation to avoid postoperative complaint and introducing further local pathology. Patients with RPD in the present study underwent surgical corrections, including tibial tuberosity osteotomy, medial patellofemoral ligament reconstruction, and lateral retinacular release. Since this study was a pilot study to propose 3D measurements and analyze the feasibility, the current data was insufficient to draw specific conclusions related to surgical outcomes. We planned to conduct a 5- to 10-year follow-up to observe the relationship between 3D measurements and prognosis, in order to explore more deeply the application of 3D measurements in clinical practice.

When analyzing the effect of WB status on patellar position, this study found that the patella was more laterally and anteriorly displaced, at a lower height, less posterior tilt and less coronal lateral tilt in the WB condition compared to the NWB condition (Fig. 5b). Previous articles investigating the effect of WB status on patellofemoral alignment were based on 2D parameters. In this study we quantified the 3D shift and tilt of the patella under WB status and established reference ranges of 3D measurements. More lateral displacement in the present study was consistent with the study of Hansen et al [31]. More lateral patellar shift might be attributed to the active quadriceps contraction under loading [6, 7].

As mentioned above there are a number of 2D parameters for quantifying patellar height, and the value of each 2D parameter is influenced by measurement methods. In the study of Pfitzner T et al [32], the modified Insall-Salvati index slightly increased while the Caton-Deschamps index significantly decreased in the WB condition compared to the NWB condition, which proved once again the importance of using standardized 3D measurements to replace multiple 2D parameters. Our results showed that, compared to the NWB condition, the patellar height decreased in the physiologic WB condition. Hansen et al found no significant difference in patellar height between weight-bearing MRI and non-weight-bearing MRI [31]. While a significant increase in patellar height was found in a study of weight-bearing DR performed with the knee flexed at 30° [33]. Differences in examination devices, knee flexion angles, and measurement methods between studies can lead to controversy over the quantification of patellar height. It emphasized the importance of using standard measurement methods based on examinations reflecting the functional status of the patellofemoral joint in future studies.

We specially compared the patellofemoral gap between WB and NWB conditions by the parameter *Y*_tilt_, which has been overlooked in previous studies. We hypothesized that the increased patellofemoral gap under the WB condition was due to the posterior displacement of the femur in the standing position [34].

When it came to the effect of WB condition on patellar tilt, we analyzed that the effect of quadriceps contraction in the sagittal plane decreased the patellar posterior tilt, and the effect of vastus medialis obliquus decreased the patellar lateral tilt in the coronal plane [5]. In conclusion, significant differences in patellofemoral alignment between the WB and NWB conditions above raised questions about surgical decisions based on conventional NWB parameters that may not truly reflect patellofemoral joint alignment. It revealed the importance of preoperative WB examinations to provide accurate preoperative planning for patellar dislocation patients and avoid postoperative complaint.

It’s worth noting that *Z*_shift_ and *Y*_tilt_ were significantly higher in the RPD group than in the control group in the WB condition but not in the NWB condition. It revealed that the recognition of abnormalities in patellar height and coronal tilt was more sensitive under the WB condition. Thus WB examination for patellofemoral joint played a role in detecting possible risk factors.

Moreover, the diagnostic performance for RPD of 3D measurements was analyzed. The results showed that *Z*_tilt_ had the best diagnostic performance for RPD in both WB and NWB conditions (AUC = 0.887 and 0.885); thus, abnormal axial lateral tilt of the patella needed to be focused on.

Our study had several limitations. First, this study did not design a comparison of 3D measurement methods with 2D measurement methods, and we are looking for 2D parameters that can stably reflect the shift and tilt of the patella in the coronal plane. Second, manual calibration of landmarks cannot avoid bias, and further studies can be conducted based on deep learning for more accurate and efficient automatic identification of landmarks. Third, the 3D measurement method has not yet been applied to the clinic to get feedback on the results, and we will combine this method with surgery and perform follow-ups.

## Conclusion

The 3D measurement method reliably and comprehensively reflected the relative spatial position relationship of the patellofemoral joint. It can be applied to the 3D preoperative planning of patellofemoral procedures. In addition, patellofemoral evaluation under the WB condition was essential to detect subtle underlying risk factors for RPD, with axial lateral patellar tilt being the best predictor.

## Supplementary information

ELECTRONIC SUPPLEMENTARY MATERIAL

## Data Availability

The datasets used and/or analyzed during the current study are available from the corresponding author upon reasonable request.

## Abbreviations

LFC: Lateral femoral condyle
MFC: Medial femoral condyle
NWBCT: Non-weight-bearing computed tomography
PIP: Patellar inferior point
PLP: Patellar lateral point
PMP: Patellar medial point
POD: Patellar oblique diameter
PSP: Patellar superior point
PTD: Patellar transverse diameter
RA: Roman arch
RPD: Recurrent patellar dislocation
WBCT: Weight-bearing cone-beam computed tomography
