# Supplementary material for: Analysis of spatial patellofemoral alignment using novel three-dimensional measurements based on weight-bearing cone-beam CT
Source: Insights Imaging. 2025 Jan 2;16:1. doi: 10.1186/s13244-024-01883-6 (PMC11695510; doi:10.1186/s13244-024-01883-6)
Supplement: Supplementary file 1 — ELECTRONIC SUPPLEMENTARY MATERIAL [file 13244_2024_1883_MOESM1_ESM.pdf]

Analysis of spatial patellofemoral alignment using novel three-dimensional measurements based on weight-bearing cone-beam CT

ELECTRONIC SUPPLEMENTARY MATERIAL

Supplementary material 1. Intra- and Inter-observer reliability

	Inter-observer				Intra-observer			
	ICC	Confidence Interval 95%		*p*	ICC	Confidence Interval 95%		*p*
		Lower	Upper			Lower	Upper	
X_{shift}	0.768	0.545	0.882	<0.001	0.894	0.738	0.952	<0.001
Y_{shift}	0.981	0.964	0.990	<0.001	0.890	0.607	0.957	<0.001
Z_{shift}	0.826	0.653	0.912	<0.001	0.942	0.885	0.971	<0.001
X_{tilt}	0.811	0.628	0.904	<0.001	0.883	0.759	0.942	<0.001
Y_{tilt}	0.913	0.819	0.957	<0.001	0.844	0.692	0.921	<0.001
Z_{tilt}	0.945	0.885	0.973	<0.001	0.962	0.925	0.981	<0.001

Supplementary 2. Diagnostic ability of 3D measurements for RPD

	WB				NWB				
	AUC (95%CI)	Cutoff Value	Sensitivity (%)	Specificity (%)	AUC (95%CI)	Cutoff Value	Sensitivity (%)	Specificity (%)	*p* value
X_{shift}	0.768 (0.691, 0.844)	3.99	71.4	74.2	0.677 (0.570, 0.784)	3.335	52	91.4	0.179
Y_{shift}	0.698 (0.615, 0.781)	39.17	55.6	78.4	0.679 (0.577, 0.781)	35.51	78	60.3	0.772
Z_{shift}	0.619 (0.528, 0.709)	11.38	54	71.1	0.592 (0.482, 0.702)	13.6	56	67.2	0.714
X_{tilt}	0.682 (0.595, 0,770)	11.36	55.6	76.3	0.625 (0.519, 0.731)	16.376	84	37.9	0.417
Y_{tilt}	0718 (0.638, 0.797)	0.19	71.4	63.9	0.598 (0.492, 0.705)	3.065	36	82.8	0.080
Z_{tilt}	0.887 (0.828, 0.946)	14.14	79.4	87.6	0.885 (0.822, 0.947)	11.815	86	77.6	0.959

AUC area under the receiver operating characteristic curve, *NWB* non-weight-bearing, *RPD* recurrent patellar dislocation, *WB* weight-bearing
